# Therapeutic Impact of Mitochondrial Transplants for Cardiovascular Diseases

**DOI:** 10.3390/ijms27094018

**Published:** 2026-04-30

**Authors:** Konstantina Antoniadou, Ioannis Shiammoutis, Christina Piperi

**Affiliations:** Department of Biological Chemistry, Medical School, National and Kapodistrian University of Athens, 11527 Athens, Greece; smg2400034@uoa.gr (K.A.); smg2400026@uoa.gr (I.S.)

**Keywords:** mitochondrial transplantation, mtDNA, CVD, ischemia/reperfusion injury, mitochondrial replacement therapy

## Abstract

Mitochondria are vital organelles for human cells with fundamental roles in major metabolic processes such as calcium homeostasis, ATP production, apoptosis and signal transduction. Defective morphology and activity of these organelles have been tightly associated with the pathological onset of severe human disorders, including cardiovascular diseases. Targeting mitochondrial dysfunction has been an area of extensive research encompassing several approaches ranging from pharmacological agents to mitochondrial replacement techniques. Among them, mitochondrial transplantation has been a rapidly evolving approach, especially in the field of cardiovascular dysfunction for the restoration of injured or damaged myocardial cells. Various methods including tunneling nanotubes, nanoblade and “mitopunch” ensure the effective mitochondrial transfer from the donor to the recipient cell, with the internalization of the organelles, via endocytosis, enabling functional restoration. Results of preclinical and clinical trials involving mitochondrial transfer support the application of this technique in improving the function of the myocardium after damage caused by ischemia reperfusion injury. Herein, we discuss the beneficial role of mitochondrial transplantation in cardiovascular diseases and the current technical challenges of mitochondrial isolation, preservation, and targeted delivery, as well as their role in advancing precision medicine, offering a patient tailored therapeutic approach.

## 1. Introduction

The mitochondria are double enveloped intracellular organelles broadly characterized as the “powerhouse of the cell”. They consist of an outer membrane being freely permeable to ions and small molecules and an inner membrane highly selective with special characteristics to enable the energy production process. The multiple “cristae” of the inner membrane increase the surface area maximizing ATP production, during oxidative phosphorylation. Mitochondria have a fundamental role in cell metabolism, especially in the energy-intensive myocardium, where these organelles are largely populated, occupying approximately one third of the volume of cardiac cells [1,2]. Moreover, it is the site of the electron transport chain (ETC), where multiple enzyme complexes and carrier proteins are involved in electron transfer, and ion pumping to the intermembrane space, ultimately leading to energy generation [1,2].

**Figure 1:**
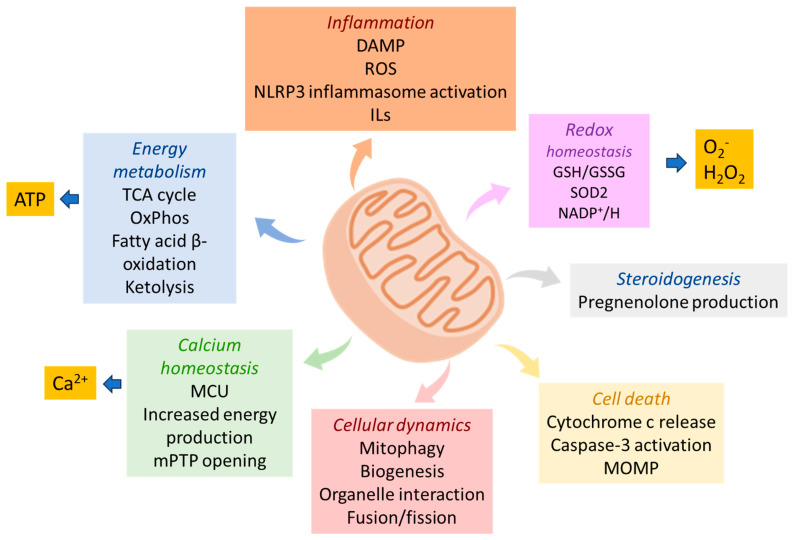
Schematic overview of main mitochondrial functions. Mitochondria are considered as major metabolic organelles orchestrating energy metabolism, being the site of vital processes such as tricarboxylic acid (TCA) cycle, oxidative phosphorylation (OxPhos), β-oxidation and ketolysis, ultimately leading to ATP production. Some OxPhos byproducts such as superoxide anion radicals (O2•^−^) and hydrogen peroxide (H2O2) are detoxified in mitochondria through the antioxidant proteins, glutathione (GSH/GSSG), superoxide dismutase 2 (SOD2) and nicotinamide adenine dinucleotide phosphate (NADP+/H), contributing to the reduction-oxidation (redox) homeostasis. Mitochondria further regulate calcium homeostasis which is associated with opening of the mitochondrial permeability transition pore (mPTP) in the membrane, the subsequent release of cytochrome c in the cytosol and the induction of apoptosis. They are also involved in the regulation of overall cellular dynamics, constantly fusing, dividing and interacting with other organelles such as endoplasmic reticulum, nucleus and lysosomes as well as participating in steroidogenesis through the production of the steroid precursor, pregnenolone. Finally, reactive oxygen species (ROS) and mitochondrial damage-associated molecular patterns (DAMPs) can activate the inflammasome and induce interleukins (IL-1β) release, implicating mitochondria in inflammation.

The increased functional properties of mitochondria are mainly attributed to their specific double-stranded DNA (mtDNA) that enables their self-replication and protein synthesis, regulating calcium signaling and apoptotic processes [1,2]. Upon elevation of calcium concentration, the mitochondrial calcium uniporter (MCU) enables its uptake by cardiomyocytes and contributes to calcium homeostasis, preventing hyperactivation and dysregulation of calcium-dependent enzymes involved in ROS production and cellular signaling pathways [3]. The calcium ions entry activates the pyruvate dehydrogenase complex and multiple dehydrogenase enzymes of the citric acid cycle, which are associated with ATP production increasing ATP levels [4]. During apoptosis, mitochondria are implicated in the activities of the intrinsic pathway, which involves the BCL-2 family proteins, BAX and BAK. Upon the formation of the BAX-BAK heterodimers, the mitochondrial pores release cytochrome c and other proteins in the cytoplasm which result in the assembly of the apoptosome and the initiation of the caspase pathway [5] (Figure 1).

The major functional impact of mitochondria in human health is illustrated by their pivotal contribution to several cardiovascular diseases (CVDs) including ischemic cardiomyopathy, heart failure, and drug-induced cardiotoxicity [6,7]. For instance, in ischemia/reperfusion injury (IRI), the characteristic mitochondrial damage is often associated with swelling and calcium overload, release of cytochrome c from the opening of the mitochondrial permeability transition pore (MPTP) and oxidative stress [8]. Altogether, these events compromise dramatically cell viability and the post-ischemic functional recovery of the myocardium.

Therefore, therapeutic strategies that target mitochondrial function or restore their function have emerged. Mitochondrial transplantation presents an innovative and most promising approach because of its significant potential to amend the previously mentioned irreversible consequences of CVD, since it can collectively modulate several aberrant biological mechanisms and overcome the challenges of single-target therapies. A number of pre-clinical studies have been conducted showing the efficacy and the advantages of this procedure as well as addressing several challenges and limitations [1,7,8].

In this review, we describe the mechanisms and methodologies currently employed in vitro and in vivo to achieve efficient mitochondrial transplantation, with emphasis on its therapeutic impact but also on the associated challenges and ethical complications. Focusing on CVD, we provide a critical evaluation of the multiple emerging mitochondrial transplantation technologies, aiming to transform traditional symptom-oriented diagnosis and treatment to a personalized approach.

## 2. Historical Background and Conceptual Foundation

Regarding the historical background of these organelles which have a uniparental inheritance, multiple discoveries have been established that demonstrated their necessity in living organisms. The first clinical implication of mitochondrial impact was the discovery of Leber hereditary optic neuropathy by the ophthalmologist Theodor Leber in 1871. His work successfully associated the hallmark manifestations of this maternally inherited disease, including bilateral optic nerve atrophy and consequent vision loss—with specific point mutations in mtDNA [9,10]. Mitochondrial dysfunction was originally connected to disease pathogenesis during the characterization of Luft disease in 1962 [11]. It was the first disorder attributed to loose coupling of oxidative phosphorylation in skeletal muscle, linked with excessive heat production and ATP depletion. Other mitochondrial abnormalities included tightly packed inner membrane ‘cristae’, and an abnormally large mitochondrial size [11]. Following these original observations, a vast range of diseases have been attributed to mitochondrial dysfunction including neurodegenerative disorders such as Alzheimer’s and Parkinson’s diseases, metabolic and cardiometabolic conditions such as type 2 diabetes and metabolic syndrome, cardiovascular diseases, and various cancers [12,13,14]. Experimental data demonstrate that impaired mitochondrial quality control and defective oxidative phosphorylation may play a pathogenic role, contributing to a wide range of human diseases [15,16,17,18]. They have urged the development of therapeutic approaches, aiming to restore mitochondrial activity such as pharmacological modulators, mitochondrial replacement and transplantation. These strategies aim to mitigate the bioenergetic deficits and minimize oxidative stress in order to improve cellular homeostasis for a range of conditions such as inherited mitochondrial disorders complex metabolic, neurodegenerative and cardiovascular diseases [19].

The mitochondrial replacement therapy (MRT) came to the surface as a way of preventing the transfer of mitochondrial diseases from the mother to her offspring. This technique focused on replacing the mother’s mtDNA which was abnormal with a donor’s healthy mitochondrial DNA. In order to perform MRT, several techniques including pronuclear transfer (PNT), maternal spindle transfer (MST) and polar genome transfer have been used [20]. The pronuclear transfer is a post-fertilization method, where two zygotes (one of the biological parents and the other of the donor) are grown in vitro. During the procedure, the parental pronuclei are inserted to the donor’s zygote containing healthy mitochondria which is then transferred to the biological mother. The maternal spindle transfer technique, unlike the PNT takes place before fertilization, and involves the removal of the spindle complex at the stage known as metaphase, positioned in the donor’s egg perivitelline space with healthy mitochondria. The embryo is then transplanted to the mother’s womb. Furthermore, the polar body genome transfer is the newest and preferred method, due to the polar body’s small size and the limited number of scarce mitochondria which minimize the chances of an mtDNA carryover [21,22,23].

Subsequent investigations demonstrated that the transfer of healthy mitochondria can restore mitochondrial function and ameliorate dysfunction in cells and tissues compromised by disease [24,25,26,27]. In 2004, the study by Rustom et al. reported the discovery of tunneling nanotubes (TNTs) which are extremely thin, dynamic membrane structures, serving as bridges between cells that enable transfer of mitochondria between healthy and UV-treated rat PC12 cells to prevent apoptosis [24]. This finding was followed by subsequent research highlighting the impact of mitochondrial transfer on regenerative medicine. In this study, a co-culture system was used to observe the transfer of mitochondrial DNA from donor mesenchymal stem cells into human lung carcinoma A549 cells lacking mitochondria. The transfer was initiated by physiological cues from the microenvironment of the damaged cells and was demonstrated to be essential for rescuing mitochondrial function, contributing to the therapeutic improvement of multiple conditions, such as cardiac myopathies [25].

Additional investigations demonstrated the role of freshly isolated autologous mitochondria in ischemic myocardium through direct injection and reported rapid internalization of mitochondria followed by improved cell viability and ATP production [26]. In rodents with cardiac ischemic reperfusion injury, mitochondrial transfer was shown to induce significant functional recovery, highlighting the need of further investigation in larger animals. In a porcine ischemia model, the direct intramyocardial injection of autologous mitochondria improved significantly the contractile function and reduced the infarct size, indicating a major translational impact [27].

To this end, another important mechanism underlying the therapeutic effects of mitochondrial transplantation is the capacity to mitigate mitochondrial calcium overload which drives cellular injury. In pathological conditions such as myocardial ischemia reperfusion, cytosolic Ca^2+^ accumulates driving mitochondrial Ca^2+^ uptake which results in the opening of the mPTP, disrupting the membrane potential and initiating necrosis and apoptosis [28]. Therefore, in this context, the transplanted mitochondria can function as bioenergetic and ionic buffers by sequestering the excess Ca^2+^ and reducing the burden on endogenous stressed mitochondria [28]. A study on dystrophic skeletal muscles has demonstrated that mitochondrial transplantation can be effective in reducing the Ca^2+^ overload and decrease pathological calcification. It can also be applied to cardiac pathologies which are characterized by Ca^2+^ dysregulation associated to injury progression [29]. In this way, the ability of transplanted mitochondrial to act as calcium scavengers is particularly significant for cardioprotection, complementing the above-mentioned roles of ATP levels restoration and oxidative stress reduction [26].

Altogether, these original and subsequent fundamental studies highlighted the pivotal role of mitochondria in normal physiology and disease, providing a robust conceptual framework for mitochondrial replacement and transplantation strategies to be developed for various clinical conditions.

## 3. Mechanisms and Methodologies of Mitochondrial Transplantation

### 3.1. Isolation, Functional Evaluation and Preparation of Donor Mitochondria

Several methods can be employed for effective mitochondrial isolation including differential centrifugation, density gradient centrifugation, and magnetic or affinity-based separation techniques [30,31,32]. Among them, differential centrifugation is the most common. During this process, the organelles are separated based on their shape, size and density. In the cell suspension, first the nuclei and cytoskeletal elements are separated followed by the mitochondria and lysosomes at higher centrifugal speeds. Other centrifugation techniques, such as Ficoll–sucrose density gradient centrifugation, have also been employed. In this method, organelles migrate to positions within the gradient that correspond to their buoyant density, enabling more precise separation [28,30]. Unfortunately, these techniques could not simultaneously achieve both a high yield and a high purity, leading to another separation technique coming to the surface, known as fractionated mitochondrial magnetic separation (FMMS) [30]. In this method, microbeads conjugated to a monoclonal anti-TOM22 antibody, are capable of binding to the mitochondrial import receptor protein subunit (TOM22) which is located on the outer membrane. Upon successful mitochondria labeling, they are placed onto a magnetic activated cell sorting (MACS) separation column, allowing for their isolation in the magnetic field [31].

The structure of the isolated mitochondria, antibody identification and enzymatic activity can be detected by transmission electron microscopy, to ensure the absence of contamination and purity. Ongoing research has shown that several sources can be used for isolation of healthy mitochondria, that can be effectively transplanted into the existing mitochondrial network of dysfunctional tissues and cells. Regarding cellular sources, cardiomyocytes, mesenchymal stem cells, platelets, and hepatocytes can be employed for mitochondrial extraction whereas tissue sources encompass muscle, adipose tissue, placenta, and liver [32].

Mitochondrial viability is evaluated by recording of the membrane potential, measured by fluorescence, as well as ATP levels and oxygen consumption rates (OCRs) using the Clark-type electrodes [33]. The main indicator of the functional integrity of isolated mitochondria is the estimation of mitochondrial membrane potential (ΔΨm), reflecting the electrochemical proton gradient across the inner mitochondrial membrane that drives ATP synthesis. It can be evaluated with the use of potential-sensitive fluorescent dyes TMRE, JC-1 or TMRM. Incubation of isolated mitochondria with the fluorescent dye enables their analysis by flow cytometry or fluorescence microscopy with appropriate controls like FCCP that can induce complete depolarization. The results are usually expressed as red/green fluorescence intensity ratios (for example, JC-1 red/green aggregate-to-monomer ratio). Functional mitochondria usually have a high proportion of polarized organelles exhibiting ≥80–90% ΔΨm-positive population. A polarization ≥70% is considered acceptable while ~60% demonstrated significant depolarization and poor activity. High red/green fluorescence ratios (>2–3) correspond to intact membrane potential and efficient proton pumping [33,34].

Regarding their capacity of ATP production, the typical threshold for high quality mitochondria is considered ≥70–80% of ATP production compared to fresh, non-isolated controls while acceptable for transplantation are also considered ≥50–60% of control levels. Less than 50% is considered poor quality and is not recommended. The standardized method is the bioluminescent luciferase-based ATP assay which quantifies ATP by measuring light emitted by the luciferase-catalyzed oxidation of luciferin in the presence of ATP, after the supply of pyruvate/malate or succinate and ADP as mitochondrial substrates. The results are normalized to protein content [35].

The mitochondrial respiratory function is measured by the OCR which reflects the ETC activity and the efficiency of oxidative phosphorylation, using the Seahorse XF analyzer or the Clark-type oxygen electrode [33,36]. The method involves the sequential addition of mitochondrial substrates (pyruvate/malate or succinate) and ADP to induce state 3 respiration, oligomycin to stimulate state 4, FCCP for uncoupling and reveal of maximal respiration and antimycin A or rotenone to enable detection of non-mitochondrial oxygen consumption. A high respiratory control ratio (RCR, stage 3/stage 4) of approximately ≥7–10 for highly active preparations is exhibited by functional mitochondria, while ≥5 is considered as acceptable for transplantation mitochondria, 3–5 is marginal and <3 refers to poor or damaged mitochondrial function. The robust ADP-stimulated increases in OCR and the low proton leak are good indicators of high-quality mitochondrial preparations to be used for transplantation [35].

Overall, functionally active mitochondria are considered those that preserve membrane potential, generate adequate ATP in response to ADP demand, maintain efficient oxidative phosphorylation and tight coupling with ATP synthesis.

### 3.2. Dosing and Delivery/Transfer Methods

Studies have shown that the dose of administered mitochondria can vary among tissues, exhibiting most commonly a dose-dependent effect which is not always linear but rather context-dependent. In cardiac, renal, neural and skeletal models, a positive relationship has been observed between mitochondrial dose and functional recovery but to a certain threshold. For cell culture delivery usually 5–50 μg of mitochondrial protein is administered per well but for cardiac tissues, typical dosing patterns are ~10^7^–10^9^ mitochondria per gram or per injection site while for neuronal tissues ~10^6^–10^8^, depending however on the route of administration: systemic or intracerebral [26].

Increasing doses of autologous mitochondria have been shown to reduce infarct size and improve cardiac contractility in myocardial ischemia models, but the benefits reached a plateau beyond optimal levels [26,32]. The same has been observed in neural injury studies where excessive mitochondria did not improve neuronal survival further [37]. This is possible due to cellular uptake capacity reaching a saturation limit or intracellular trafficking restriction. Therefore, the relationship is non-linear and mitochondrial transplantation seems to follow a dose-dependent therapeutic effect up to a certain threshold that is specific for each tissue.

The recently developed artificial mitochondrial transfer using the photothermal nanoblade has been shown to enable effective delivery, circumventing the need for cell fusion or endocytosis [38]. This procedure relies on a photo-absorptive titanium tip that is mounted on a microcapillary pipette which is positioned in gentle contact with the plasma membrane. A pulsed 532 nm laser is then focused on the pipette tip, generating a transient cavitation bubble which under pressure, allows for the direct transfer of large cargo, such as mitochondria into the target cells. Based on this technology, a pressure assisted transfer device called “Mitopunch” was developed, which enables simultaneous delivery of healthy mitochondria to multiple recipient cells.

The mitochondria can also be transferred to recipient cells via microinjection, to ensure targeted delivery. Liposomes, artificial spherical vesicles resembling the composition of the plasma membrane, have been extensively used for this process. They enclose the mitochondria obtained from donor cells, protect them from external conditions and allow for a localized delivery [39].

**Figure 2:**
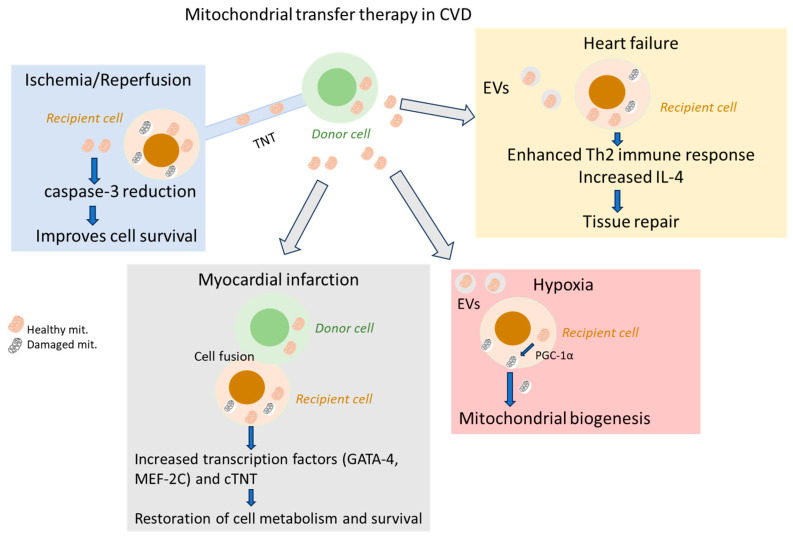
Different pathways of mitochondrial transfer between donor cells and cardiac cells with damaged mitochondria. Pre-clinical studies CVD models have shown that the transfer of mitochondria from donor cells to cardiac cells (cardiomyocytes) can be achieved through several different ways including tunneling nanotubes (TNTs), cell fusion and extracellular vesicles (EVs) [38,39,41,42,43,44,45,46]. In ischemia and reperfusion cases, healthy mitochondria can be transferred to stressed or damaged cardiomyocytes through TNTs, enhancing their survival through reduction in caspase-3. In myocardial infarction models, donor cells can transiently or permanently fuse cardiomyocytes to exchange intracellular components and mitochondria. Mitochondrial transfer increases the levels of transcription factors Myocyte Enhancer Factor 2C (MEF-2C), GATA Binding Protein 4 (GATA-4), and cardiac troponin T (cTnT), restoring cell metabolism and survival of damaged cells [28]. In hypoxia and heart failure, EVs from donor cells have been shown to induce mitochondrial biogenesis mediated by peroxisome proliferator-activated receptor gamma coactivator 1-alpha (PGC-1α) while macrophage-derived mitochondria increase circulating levels of interleukin 4 (IL-4) and promote Th2 responses, increasing heart function.

Tunneling nanotubes (TNTs), a natural delivery method composed of F-actin and microtubules, have also been implemented in mitochondrial transfer which consists of tubular cytoplasmic extensions originating from the cell membrane. With a width ranging from 50 to 1500 nm and a length from 5 to 120 μm, TNTs can breach the gap between long -distance cells via unidirectional transport of cellular contents [40]. The formation of TNTs involves several proteins including small GTPases, TNF-α inducible protein 2, and leukocyte-specific transcript 1 and can be modulated by caspase-3 activation, which is associated with the early release of cytochrome c from mitochondria [41]. The transfer of mitochondria through TNTs has been shown to be associated with the mitochondrial Rho small GTPase 1 (MIRO) [42]. A different mitochondrial transfer methodology involves cell fusion of two or more cells, which facilitates the intercellular movement of mitochondria and cellular components, usually triggered by injury and inflammation or chemical exposure [24,43,44]. Co-culture of mesenchymal stem cells with cardiomyocytes has been shown to induce partial fusion, enabling mitochondrial transfer (Figure 2) [45].

Extracellular vesicles (EVs) have also been reported to carry mitochondria as well as mitochondrial components such as DNA (mtDNA) and proteins, influencing the recipient cell’s function. A study has shown that healthy mesenchymal stem cells are capable of packaging functional mitochondria into EVs and release them to damaged cells, improving the recipient’s energy production. The release mechanism is often calcium-dependent, involving signaling pathways such as the CD38 and inositol trisphosphate receptor (IP3R) signaling (Figure 2) [46].

### 3.3. Integration and Functional Restoration

Mitochondrial internalization depends on the integrity of the outer membrane and its associated proteins. Different cell type-specific uptake mechanisms exist, including macropinocytosis, actin-dependent endocytosis, and caveola- and clathrin-mediated endocytosis. In the case of macropinocytosis, Kitani et al. demonstrated its essential role using specific inhibitors. Treatment of endometrial mesenchymal stem cells (EMCs) with the Na^+^/H^+^ exchanger inhibitor 5-(N-ethyl-N-isopropyl)amiloride (EIPA) induced a strong reduction in mitochondrial uptake, as analyzed by flow cytometry and fluorescence microscopy [47]. However, actin-dependent endocytosis is mostly recognized as the primary mechanism for mitochondrial internalization. The continuous polymerization and depolymerization of actin filaments enable the formation of membrane invaginations and the subsequent detachment of vesicles from the cytosolic side of the plasma membrane. Cytochalasin D-induced inhibition of actin dynamics prevents F-actin from binding to cofilin and slows G-actin conversion to F-actin, reducing significantly the mitochondrial uptake. Moreover, actin is required for caveola- and clathrin-mediated endocytosis, contributing to multiple stages of clathrin-coated vesicle formation. Of note, pre-incubation of cardiomyocytes with methyl-β-cyclodextrin, a cyclic oligosaccharide that can disrupt lipid raft organization and increase membrane fluidity, did not affect mitochondrial internalization, indicating that other actin-dependent mechanisms are predominantly responsible for mitochondrial uptake in these cells [48]. The intracellular dynamics of mitochondria in human induced pluripotent stem cell-derived cardiomyocytes (iPS-CMs) and human cardiac fibroblasts (HCFs) have been investigated using three-dimensional super-resolution structured illumination microscopy (3D SR-SIM) and transmission electron microscopy (TEM). In these studies, labeling of mitochondria was performed with either gold nanoparticles or fluorescent proteins. Specific cellular compartments in iPS-CMs and HCFs were also fluorescently marked, enabling the tracking of mitochondrial movement through the endolysosomal system and their functional integration into the host mitochondrial network [49].

## 4. Preclinical Evidence of Mitochondrial Translation Benefits in Cardiovascular Diseases

### 4.1. In Vitro Studies

In vitro studies of mitochondrial uptake have been conducted using co-cultures of rat neonatal cardiomyocytes (rCMs) labeled with MitoTracker Green and human adipose-derived stem cells (hADSCs) labeled with MitoTracker Red. The co-cultures were exposed to hypoxic conditions (1% O_2_) for 24 h to mimic cardiac ischemia. Following this, cells were immunostained with anti-cardiac actinin and anti-human mitochondria antibodies to visualize the transfer of mitochondria from hADSCs to rCMs [50]. Similarly, Rossi et al. demonstrated that healthy mitochondria isolated from renal proximal tubule cells could be co-incubated with injured recipient cells, resulting in their integration and functional recovery. Observed effects included increased ATP production, enhanced proliferative capacity, significant reduction in reactive oxygen species, and decreased cytotoxicity [51].

### 4.2. In Vivo Studies

Several animal studies have demonstrated functional benefits of mitochondrial transfer in the myocardium (Table 1) [52,53,54,55]. In the study by Blitzer et al., Yorkshire pigs were subjected to 30 min of ischemia through temporary occlusion of the left anterior descending artery to evaluate the efficacy of delayed mitochondrial transfer [52]. One group of pigs received autologous mitochondria isolated from skeletal muscle, while the other received only the vehicle as a control. The mitochondria or vehicle were delivered as a bolus into the left coronary ostium.

Mitochondria-treated pigs showed significant improvements in left ventricular ejection fraction (EF) and the percentage change in left ventricular diameter from diastole to systole. At 4 h post-delivery, treated animals exhibited an increased fractional area change, indicating enhanced myocardial contractility following injury. Moreover, infarct size was markedly smaller in the mitochondria-treated pigs compared with controls [52,53]. New Zealand rabbits have also been used in a pivotal study by Cowan et al. to assess the protective effects of exogenous mitochondria on ischemic myocardium. The mitochondria were delivered via the coronary vasculature and were found in close proximity to both blood vessels and cardiomyocytes. Additionally, autologous mitochondria isolated from the liver were shown to enhance myocardial function and reduce infarct size [53,54].

In a C57BL/6J mice model of heart transplantation, isolated allogeneic mitochondria from the gastrocnemius muscle were transferred antegrade to the coronary arteries by injection to the coronary ostium. In conjunction with prolonged cold ischemic time (CIT), this treatment ameliorated graft function, reduced graft tissue injury and diminished neutrophil infiltration compared to control groups [55]. Another study investigating the efficacy of transplantation of mitochondria isolated from non-diabetic or Zucker diabetic fatty (ZDF fa/fa) rats delivered to the coronary arteries through the aortic cannula, demonstrated significantly improved functional recovery and reduced tissue damage after ischaemia reperfusion in diabetic hearts [56].

## 5. Clinical Applications and Trials

Completed preclinical and clinical studies as well as ongoing investigations aim to evaluate the therapeutic potential of mitochondrial transplantation in disorders linked to mitochondrial dysregulation (Table 2).

The first human study on mitochondrial transplantation was performed in five pediatric patients with myocardial ischemia reperfusion injury, following open-heart surgery [57]. Those patients suffered from multiple congenital defects such as hypoplastic left heart syndrome, left ventricular outflow tract obstruction, hypoplastic left heart syndrome and tricuspid atresia. Due to their myocardial ischemia reperfusion injury, they were dependent on extracorporeal membrane oxygenation (ECMO) for their survival. Autologous mitochondria were isolated from the rectus abdominis muscle of each patient, and ten injections—each containing approximately 1 × 10^7^ mitochondria—were administered into the myocardium [57]. All five patients exhibited significant improvement in ventricular function. Four patients were successfully weaned off extracorporeal membrane oxygenation (ECMO), whereas the fifth patient experienced multi-organ failure and remained dependent on ECMO support [54]. However, an important limitation of the study is the lack of a control group, introducing a potential bias in the clinical outcome interpretation, rendering it difficult to attribute the observed clinical improvements to mitochondrial transplantation, and not to other perioperative or supportive interventions.

Another randomized, triple-blinded trial involved thirty ST-elevation myocardial infarction (STEMI) patients who were divided into a control group receiving standard therapy and a treatment group [58]. Platelet-derived mitochondria from each individual patient in the treatment group were extracted and injected in their coronary arteries. Even though both groups showed an improvement in the percentage of blood pumped out of the left ventricle per heartbeat, a greater statistically significant overall improvement was observed in the treatment group after a period of 40 days (36–45.7% for the control group vs. 35.6–47.7% for the treatment group) [58,59]. However, it should be noted that this is an early-phase, single-center study with a short follow-up duration (40 days), precluding robust assessment of long-term efficacy and safety outcomes. The modest improvements observed in left ventricular ejection fraction and exercise capacity should be considered as preliminary efficacy signals rather than definitive clinical outcome. In addition, the limited cohort size and potential heterogeneity in patient characteristics may affect generalizability, indicating that larger, multicenter randomized trials with longer follow-up are required to validate its therapeutic efficacy and establish its clinical relevance.

The clinical trial (NCT02851758) conducted by the McCully group represents a widely recognized attempt at autologous mitochondria transplantation for myocardial treatment after ischemia reperfusion injury (IR). The IR injury which occurs when blood flow is restored to previously obstructed cardiac tissue, is a major contributor to adverse cardiovascular outcomes, including cardiac arrest. In this study, healthy mitochondria were isolated from the rectus abdominis of each patient and directly infused into the ischemia-damaged myocardium. Close monitoring over several days revealed improved ventricular function in the treated patients. The safety and tolerability of the procedure were further supported by the absence of systemic inflammatory response syndrome markers before or after mitochondrial injection [60].

The research study of Walker et al. is also investigating the therapeutic potential of autologous mitochondrial transplantation (NCT04998357). Their trial, currently in the patient recruitment phase, aims to evaluate, for the first time in humans, the efficacy of this approach for cerebral ischemia. Cerebral ischemia is characterized by reduced blood flow to the brain, leading to tissue injury or death (cerebral infarction or stroke) due to insufficient oxygen and nutrient delivery. In this study, mitochondria will be isolated from muscle tissue near the surgical site and delivered into the cerebral artery using a microcatheter during endovascular reperfusion treatment [61].

Several clinical trials conducted by Paean Biotechnology Inc. involve the transplantation of mitochondria derived from allogeneic umbilical cord-derived mesenchymal stem cells [62,63]. The trials target refractory cases of polymyositis (PM) and dermatomyositis (DM), which are multifactorial inflammatory diseases affecting the muscles, skin, and various other organs. These conditions are characterized by elevated muscle enzyme levels, muscle weakness, and visible skin alterations, with autoimmune mechanisms identified as the primary underlying cause. The trial involves intravenous administration of mitochondria to patients and evaluation of their safety through initial monitoring of dose-limiting toxicities (DLTs) at the first two weeks of administration [39].

## 6. Challenges and Ethical Considerations

Mitochondrial transplantation is a very promising approach for the treatment of mitochondrial and ischemic disorders, facing however several significant challenges that need to be overcome in order to be clinically applied. The most important challenge is to ensure their efficient and targeted delivery to the desired tissues. Effective ways to maintain the viability and functional integrity of isolated mitochondria during the isolation and transplantation steps need to be further explored and standardized protocols to be established. Of importance, significant efforts to avoid potential immune or inflammatory responses are highly demanded in order to achieve long-term integration into the recipient mitochondrial network for clinical applications [24,64,65,66,67,68].

The long-term fate of transplanted mitochondria remains poorly understood with available data suggesting that mitochondrial persistence and function are regulated by intracellular quality control mechanisms. Although exogenous mitochondria can integrate with the host mitochondrial network, enhancing ATP production and metabolic recovery, a significant subset may be subjected to mitophagy, especially if damaged or exposed to oxidative stress or calcium overload [69].

Gradually, the transplanted mitochondria will fuse with the endogenous ones or will be selectively degraded through autophagy, limiting their durability [69]. Additionally, the introduction of exogenous mitochondrial DNA may induce heteroplasmy with both donor and recipient mtDNA coexisting in the same cell, without knowledge of their long-term stability. Studies indicate that the beneficial function is maintained beyond the presence of transplanted mitochondria, indicating the triggering of adaptive responses [70]. It is therefore evident that longitudinal studies are demanded to clarify the longevity of therapeutic effects and the role of mitochondrial transplantation in durable cellular reprogramming.

### 6.1. Technical Limitations

Isolated mitochondria remain functionally active for only 60–120 min when stored on ice; therefore, their isolation and purification must be performed rapidly and precisely, typically using differential centrifugation, density gradient separation, and differential filtration techniques [64]. The clinical impact of mitochondrial transplantation could be further enhanced by having ready-to-use storage of already isolated and purified mitochondria, that can also be stored for an extended period of time, to enable their use in surgical settings. Optimal storage conditions present another requirement, to avoid the potential damage of the outer or inner mitochondrial membrane which could alter membrane permeability and decrease the ATP producing capacity of the organelle, respectively [64].

Recognition of the suitable healthy source which contains functional mitochondria and decision of the type of transplantation, ranging from autologous, xenogeneic or heterologous, poses a significant challenge in mitochondrial transplantation. For example, myocardial infarction requires mitochondria isolated from autologous skeletal muscle or platelets for a faster and safer intervention [71,72]. Preclinical evidence has shown that the donor’s age is an important factor for mitochondrial isolation, with younger ages preferred since older ages often present impaired oxidative phosphorylation, declined ATP production and promotion of ROS [73,74]. A study reported that out of all the potential mitochondrial donors, only 25% of them were found to be eligible due to these strict criteria [75]. Apart from these, it is also crucial to determine the optimal administration route and the number of mitochondria that can be isolated from the available cells or tissues, selecting the minimally invasive process and the easily accessible tissue [65,66]. The transplantation process is usually carried out by skilled personnel, requiring specific equipment which unfortunately cannot be found in each medical center. Moreover, not every patient will be able to cover this costly procedure that is not currently covered by multiple insurance plans.

### 6.2. Immunological Compatibility and Rejection Risks

Patients with inherited mitochondrial disorders are generally not suitable candidates for autologous mitochondrial transplantation, as their mitochondria are likely to carry genetic defects across multiple tissues, which would render the procedure ineffective [67]. This means that those patients need to be treated with allogenic mitochondria, which can sometimes induce an immune response due to activation of Toll-like receptors following recognition of DAMPs [68]. Common DAMPs include RNA, mtDNA, and bioactive metabolites such as N-formyl peptides, cytochrome c, and ATP, which can trigger immune and inflammatory responses when released. Studies have shown that mitochondria are damaged in numerous human diseases, including cardiovascular disorders [76,77]. Consequently, permeabilization of the mitochondrial double membrane can occur, leading to the release of DAMPs that may activate an immune response via inflammasome and cGAS–STING signaling pathways. In contrast, transplantation of allogeneic mitochondria has been shown to potentially attenuate immune activation, as evidenced by reduced levels of inflammatory cytokines such as IL-6, TNF-α, and hs-CRP in models of myocardial ischemia reperfusion injury. However, a study reported that purified allogeneic mitochondria can also induce an inflammatory response in vascular endothelial cells by upregulating adhesion molecules and promoting cytokine secretion [78].

Another important aspect of mitochondrial transplantation is the potential to develop adaptive immune responses, either humoral and/or T-cell mediated against mitochondrial antigens. The bacterial evolution of these organelles and their immunogenic content of mitochondrial proteins and mtDNA can be recognized as non-self in allogeneic settings. Upon uptake by APCs, mitochondrial peptides can be processed and presented by MHC molecules, potentially triggering CD4^+^ and CD8^+^ T cell activation [78,79,80]. Additionally, the exposure to extracellular mitochondrial components can stimulate the production of anti-mitochondrial antibodies. These responses may be particularly relevant upon repeated dosing or systemic administration, with prolonged antigen exposure inducing immune sensitization. Future studies are therefore needed to assess both humoral and cellular responses to characterize the immunogenic profile and the long-term safety of mitochondrial transplantation.

### 6.3. Ethical Implications

Ethical issues play a central role in mitochondrial transplantation, particularly in regard to the selection of proper donors. Important questions are raised on whether the donation should be regulated as gamete (egg) or tissue because mtDNA is transmitted through the cytoplasm [68]. Moreover, the mitochondrial donor’s genetic contribution plays a significant role since mitochondria are vital for other important processes such as energy metabolism, apoptosis, and signal transduction pathways. There is also a possibility that mitochondrial–nuclear interaction can alter nuclear gene expression through epigenetic mechanisms. Another ethical issue arises from interventions that involve germline mitochondrial replacement which although the social and genetic parents remain distinct, the potential to create the so-called “three-parent” offspring, complicates the situation.

Apart of the issues of heritable genetic modification and intergenerational risk, somatic mitochondrial transplantation is also associated with different ethical aspects, centered on the clinical application and the translational safety. Among the key issues is the adequacy of informed consent, especially when comparing autologous mitochondrial use, which minimizes immunological and ethical concerns, with allogeneic or donor-derived preparations, which introduce considerations of donor screening, biological variability, and potential immune incompatibility [68,81]. Additionally, somatic mitochondrial transplantation is still mainly experimental, raising concerns of its application outside formal clinical trials in respect to off-label use, requiring a very careful regulatory oversight to prevent premature clinical adoption.

Altogether, these considerations highlight the requirement of careful design, informed consent, and ongoing clarifications of the long-term implications of mitochondrial donation and transplantation [82].

## 7. Emerging Technologies and Future Directions

It is evident that mitochondrial transplantation represents a distinctive therapeutic strategy when compared to other emerging approaches which can target mitochondrial dysfunction, since it aims to directly restore bioenergetic capacity through the delivery of functional organelles, rather than modifying the cellular pathways or replacing cells.

The gene editing technologies (such as CRISPR-based approaches) can more precisely correct the nuclear or mitochondrial DNA defects but they still remain limited due to delivery challenges and off-target effects. Moreover, in regard to mtDNA, they face technical constraints related to heteroplasmy and editing efficiency [83].

The stem cell therapies that involve mesenchymal or induced pluripotent stem cells, can be also used to enhance tissue repair through endogenous mitochondrial transfer, paracrine signaling and immunomodulation. However, their therapeutic potential is mostly indirect, and quite variable, raising concerns regarding long-term safety [84]. In addition, pharmacological mitochondrial modulators including antioxidants, permeability transition pore inhibitors or electron transport chain activators, can be readily used but they can only provide partial or transient protection and do not restore the damaged mitochondria [85].

Novel approaches employing genetically engineered mitochondria have shown significant improvement in mitochondrial transplantation. The use of gene editing enables correction of genetic defects, taking place either in mtDNA or in the nuclear genes encoding mitochondrial proteins. Some myocardial-related preclinical studies have demonstrated the potential of this technique in mouse models [86,87,88]. Bacman et al., showed that delivery of mitoTALENS via AAV9, succeeded in replacing thymine with cytosine at position 50624 (m.5024C>T) in the mitochondrial genome, restoring normal expression of the alanine-carrying tRNA and abolishing the mitochondrial cardiomyopathy symptoms [89]. Similar results were also observed during the transfer of mitoZFNs, by the AAV9.45 delivery method in mice, which not only corrected the genetic defect but also improved metabolic compounds such as aspartate, pyruvate, and lactate levels [86].

Another emerging technology is the introduction of mutations in the mitochondrial DNA by double-stranded DNA deaminase (DddA)-derived cytosine base editor (DdCBE) [87]. A study evaluated its transfer into the heart of neonatal and adult mice by an adeno-associated virus vector and reported that it successfully achieved targeted edits in the mtDNA, suggesting that future identification and repair of pathogenic mitochondrial mutations, may be used to potentially treat previously incurable diseases [87]. Stem cells play a significant therapeutic role with regard to cell injury. These cells, after migrating to the damaged area, differentiate into the cells that were damaged, remove the toxic substance buildup by the injured cells, and release growth factors. Additionally, they transfer healthy mitochondria to the injured cells by the process of endocytosis or via the TNTs. One of the advantages of combining mitochondrial transfer with stem cell therapy is the decreased possibility of eliciting an immune response, due to the absence of antigens on the surface of mesenchymal stem cells (MSCs), enabling the patient to accept stem cell mitochondria from a different individual. The stem cells are also capable of a long-term proliferation and expansion, providing a large number of mitochondria required for clinical applications [88].

In the context of personalized medicine, mitochondrial transplantation can be tailored to each individual based on their mitochondrial and nuclear genetic background, as well as the specific mitochondrial needs of the affected tissue, in order to maximize therapeutic efficacy [32]. According to the study of Tragni et al., a personalized nutrition-based approach tailored to a patient’s mitochondrial genotype could represent an initial step toward individualized mitochondrial medicine. For example, thiamine (vitamin B1) which is a hydrophilic precursor of thiamine pyrophosphate, uses the nuclear encoded SLC25A19 carrier protein for its transport to mitochondria. In addition, it is an important cofactor for several essential enzymes, such as 2-oxoglutarate dehydrogenase and pyruvate dehydrogenase, which are vital for proper mitochondrial function. There are, however, several limitations of this approach such as the wide diversity of mitochondrial defects, challenges in delivering therapies directly to mitochondria, and the lack of reliable biomarkers [89]. Future biobanking of healthy autologous mitochondria can be a meaningful option for personalized therapies as currently performed with induced pluripotent stem cells.

## 8. Conclusions

A significant number of preclinical and clinical studies indicate that mitochondrial transplantation can effectively restore mitochondrial function in damaged tissues such as the myocardium. Long-term studies are however demanded to elucidate the underlying functional mechanisms of transplanted mitochondria, to overcome the significant challenges related to dosing, delivery, mitochondrial survival and enable their widespread clinical application. Current clinical trials aim to further evaluate the safety, efficacy and durable beneficial effects across diverse clinical settings.

## Figures and Tables

**Table 1:** Preclinical animal studies of mitochondrial transplantation in cardiovascular diseases.

Model/ Condition	Donor Source	Recipient/ Target Organ	Notes	Outcome	Reference
Porcine Myocardial Ischemia	Autologous mitochondria from skeletal muscle	Left coronary ostium (bolus delivery)	LAD artery occluded for 30 min; comparison vs. vehicle-only controls	↑ Ejection fraction, ↑ fractional area changes at 4 h, ↑ LV contraction strength, ↓ infarct size	[52]
Rabbit Myocardial Ischemia	Exogenous mitochondria and autologous liver-derived mitochondria	Coronary vasculature	Mitochondria located near blood vessels and cardiomyocytes after delivery	Improved myocardial function and ↓ infarct size	[54]
C57BL/6 J mice, Heart Transplant	Gastrocnemius muscle	ICA infusion		↑ Cold ischemic time; ↓ Graft tissue injury, neutrophil infiltration	[55]
Zucker diabetic fatty rat, warm ischemia	Skeletal muscle	ICA infusion		↑ Mitochondrial function; ↓ Infarct size, edema	[56]

↑ Increased, ↓ Decreased.

**Table 2:** Clinical studies of mitochondrial transplantation in human patients.

Condition/Disease	Donor Source	Target Organ	Phase	Notes	Status/Outcome	Reference
Pediatric Myocardial Ischemia Reperfusion Injury (Post-Open Heart Surgery)	Autologous mitochondria from rectus abdominis	Myocardium (10 injections; ~1 × 10^7^ mitochondria per dose)	Early human study	First ever human mitochondrial transplantation study; patients had congenital heart defects (HLHS, LVOTO, tricuspid atresia)	4/5 recovered sufficiently to discontinue ECMO; 1 patient remained ECMO-dependent due to multi-organ failure	[57]
ST-Elevation Myocardial Infarction (STEMI)	Autologous platelet-derived mitochondria	Coronary arteries	Control vs. treatment group	Control: 36–45.7% LVEF; Treatment: 35.6–47.7% LVEF after therapy	Both groups improved LVEF; mitochondrial-treated group showed greater improvement after 40 days	[58,59]
Ischemia reperfusion injury (IR)	Autologous mitochondria transplantation for myocardial treatment	Ischemic heart muscle	Phase I	Improved ventricular function in the treated patients	Complete	[60]
Cerebral Ischemia—(First-in-Brain Study)	Autologous muscle mitochondria (from nearby surgical site)	Brain artery via microcatheter during reperfusion	Phase 1	First human trial delivering mitochondria into the brain for ischemic stroke	Recruiting	[61]
Polymyositis/Dermatomyositis	Allogeneic mitochondria from umbilical cord-derived MSCs	Systemic IV administration	Phase 1	Monitoring dose-limiting toxicities within first 2 weeks; targeting refractory inflammatory myopathies	Ongoing	[62,63]

## Data Availability

No new data were created or analyzed in this study. Data sharing is not applicable to this article.

## References

[1] Miao X., Jiang P., Wang Z., Kong W., Feng L. (2025). Mitochondrial transplantation: A novel therapeutic approach for treating diseases. MedComm.

[2] Guan S., Zhao L., Peng R. (2022). Mitochondrial respiratory chain supercomplexes: From structure to function. Int. J. Mol. Sci..

[3] Zaglia T., Ceriotti P., Campo A., Borile G., Armani A., Carullo P., Prando V., Coppini R., Vida V., Stølen T.O. (2017). Content of mitochondrial calcium uniporter (MCU) in cardiomyocytes is regulated by microRNA-1 in physiologic and pathologic hypertrophy. Proc. Natl. Acad. Sci. USA.

[4] Kannurpatti S.S. (2017). Mitochondrial calcium homeostasis: Implications for neurovascular and neurometabolic coupling. J. Cereb. Blood Flow Metab..

[5] Yu L., Zhu G., Zhang Z., Yu Y., Zeng L., Xu Z., Weng J., Xia J., Li J., Pathak J.L. (2023). Apoptotic bodies: Bioactive treasure left behind by the dying cells with robust diagnostic and therapeutic application potentials. J. Nanobiotechnol..

[6] Ahmad S.S., Ansari J.A., Ansari T.M., Zaidi S.M.H. (2025). Mitochondrial dysfunction in cardiac diseases: Insights into pathophysiology and clinical outcomes. Curr. Cardiol. Rev..

[7] Peña-Blanco A., García-Sáez A.J. (2018). Bax, Bak and beyond—Mitochondrial performance in apoptosis. FEBS J..

[8] Baines C.P. (2009). The mitochondrial permeability transition pore and ischemia-reperfusion injury. Basic Res. Cardiol..

[9] Desnuelle C. (2003). Evolution du concept de maladie mitochondriale. Bull. Acad. Natl. Med..

[10] Piotrowska A., Korwin M., Bartnik E., Tońska K. (2015). Leber hereditary optic neuropathy. Gene.

[11] DiMauro S., Bonilla E., Lee C.P., Schotland D.L., Scarpa A., Conn H., Chance B. (1976). Luft’s disease. J. Neurol. Sci..

[12] Yang H.M. (2025). Mitochondrial dysfunction in neurodegenerative diseases. Cells.

[13] Rocca C., Soda T., De Francesco E.M., Fiorillo M., Moccia F., Viglietto G., Angelone T., Amodio N. (2023). Mitochondrial dysfunction at the crossroad of cardiovascular diseases and cancer. J. Transl. Med..

[14] Diaz-Vegas A., Sanchez-Aguilera P., Krycer J.R., Morales P.E., Monsalves-Alvarez M., Cifuentes M., Rothermel B.A., Lavandero S. (2020). Is mitochondrial dysfunction a common root of noncommunicable chronic diseases?. Endocr. Rev..

[15] Ait-Aissa K., Blaszak S.C., Beutner G., Tsaih S.W., Morgan G., Santos J.H., Flister M.J., Joyce D.L., Camara A.K.S., Gutterman D.D. (2019). Mitochondrial oxidative phosphorylation defect in coronary artery disease. Sci. Rep..

[16] Shoffner J.M., Watts R.L., Juncos J.L., Torroni A., Wallace D.C. (1991). Mitochondrial oxidative phosphorylation defects in Parkinson’s disease. Ann. Neurol..

[17] Sturm G., Karan K.R., Monzel A.S., Santhanam B., Taivassalo T., Bris C., Ware S.A., Cross M., Towheed A., Higgins-Chen A. (2023). OxPhos defects cause hypermetabolism and reduce lifespan in mitochondrial diseases. Commun. Biol..

[18] Walker B.R., Theard L.M., Pinto M., Rodriguez-Silva M., Bacman S.R., Moraes C.T. (2024). Restoration of defective oxidative phosphorylation prevents mitochondrial encephalopathy. EMBO Mol. Med..

[19] Hayashida K., Takegawa R., Endo Y., Yin T., Choudhary R.C., Aoki T., Nishikimi M., Murao A., Nakamura E., Shoaib M. (2023). Mitochondrial transplantation improves outcomes after cardiac arrest. BMC Med..

[20] Sharma H., Singh D., Mahant A., Sohal S.K., Kesavan A.K., Samiksha (2020). Development of mitochondrial replacement therapy: A review. Heliyon.

[21] Paull D., Emmanuele V., Weiss K.A., Treff N., Stewart L., Hua H., Zimmer M., Kahler D.J., Goland R.S., Noggle S.A. (2013). Nuclear genome transfer eliminates mitochondrial DNA variants. Nature.

[22] Yamada M., Akashi K., Ooka R., Miyado K., Akutsu H. (2020). Mitochondrial genetic drift after nuclear transfer. Int. J. Mol. Sci..

[23] Wang T., Sha H., Ji D., Zhang H.L., Chen D., Cao Y., Zhu J. (2014). Polar body genome transfer prevents mitochondrial disease transmission. Cell.

[24] Chen R., Chen J. (2024). Mitochondrial transfer for metabolic diseases. Front. Endocrinol..

[25] Paliwal S., Chaudhuri R., Agrawal A., Mohanty S. (2018). Regenerative abilities of MSCs through mitochondrial transfer. J. Biomed. Sci..

[26] McCully J.D., Del Nido P.J., Emani S.M. (2023). Mitochondrial transplantation: The advance to therapeutic application and molecular modulation. Front. Cardiovasc. Med..

[27] Kaza A.K., Wamala I., Friehs I., Kuebler J.D., Rathod R.H., Berra I., Ericsson M., Yao R., Thedsanamoorthy J.K., Zurakowski D. (2017). Myocardial rescue with mitochondrial transplantation. J. Thorac. Cardiovasc. Surg..

[28] Huang Y., Li W., Sun H., Guo X., Zhou Y., Liu J., Liu F., Fan Y. (2024). Mitochondrial transfer in the progression and treatment of cardiac disease. Life Sci..

[29] Dubinin M.V., Stepanova A.E., Mikheeva I.B., Igoshkina A.D., Cherepanova A.A., Talanov E.Y., Khoroshavina E.I., Belosludtsev K.N. (2024). Reduction of Mitochondrial Calcium Overload via MKT077-Induced Inhibition of Glucose-Regulated Protein 75 Alleviates Skeletal Muscle Pathology in Dystrophin-Deficient mdx Mice. Int. J. Mol. Sci..

[30] Hubbard W.B., Harwood C.L., Prajapati P., Springer J.E., Saatman K.E., Sullivan P.G. (2019). Fractionated mitochondrial magnetic separation. Sci. Rep..

[31] Hornig-Do H.T., Günther G., Bust M., Lehnartz P., Bosio A., Wiesner R.J. (2009). Isolation of functional pure mitochondria by superparamagnetic microbeads. Anal. Biochem..

[32] Wang X., Liu Z., Zhang L., Hu G., Tao L., Zhang F. (2024). Mitochondrial transplantation for the treatment of cardiac and noncardiac diseases. Life Med..

[33] Silva A.M., Oliveira P.J. (2018). Evaluation of respiration with Clark-type electrode in isolated mitochondria. Methods Mol. Biol..

[34] Perry S.W., Norman J.P., Barbieri J., Brown E.B., Gelbard H.A. (2011). Mitochondrial membrane potential probes and the proton gradient: A practical usage guide. Biotechniques.

[35] Spinazzi M., Casarin A., Pertegato V., Salviati L., Angelini C. (2012). Assessment of mitochondrial respiratory chain enzymatic activities on tissues and cultured cells. Nat. Protoc..

[36] Plitzko B., Loesgen S. (2018). Measurement of Oxygen Consumption Rate (OCR) and Extracellular Acidification Rate (ECAR) in Culture Cells for Assessment of the Energy Metabolism. Bio Protoc..

[37] Gollihue J.L., Patel S.P., Eldahan K.C., Cox D.H., Donahue R.R., Taylor B.K., Sullivan P.G., Rabchevsky A.G. (2018). Mitochondrial transplantation restores respiration, reduces cell death, and improves locomotor recovery after spinal cord injury. J. Neurotrauma.

[38] Wu T.H., Sagullo E., Case D., Zheng X., Li Y., Hong J.S., TeSlaa T., Patananan A.N., McCaffery J.M., Niazi K. (2016). Mitochondrial transfer by photothermal nanoblade restores metabolite profile. Cell Metab..

[39] Kim H.R., Cho H.B., Lee S., Park J.I., Kim H.J., Park K.H. (2023). Fusogenic liposomes encapsulating mitochondria for osteoarthritis therapy. Biomaterials.

[40] Austefjord M.W., Gerdes H.H., Wang X. (2014). Tunneling nanotubes: Diversity in morphology and structure. Commun. Integr. Biol..

[41] Qin Y., Jiang X., Yang Q., Zhao J., Zhou Q., Zhou Y. (2021). Functions and mobility of mitochondrial transfer. Front. Oncol..

[42] Las G., Shirihai O.S. (2014). Miro1: New wheels for mitochondrial transfer. EMBO J..

[43] Acquistapace A., Bru T., Lesault P.F., Figeac F., Coudert A.E., le Coz O., Christov C., Baudin X., Auber F., Yiou R. (2011). Human mesenchymal stem cells reprogram adult cardiomyocytes toward a progenitor-like state through partial cell fusion and mitochondria transfer. Stem Cells.

[44] Hu C., Shi Z., Liu X., Sun C. (2024). Mitochondrial transplantation research progress. Int. J. Mol. Sci..

[45] Ma Z., Yang H., Liu H., Xu M., Runyan R.B., Eisenberg C.A., Markwald R.R., Borg T.K., Gao B.Z. (2013). MSC–cardiomyocyte interactions via mitochondrial transfer. PLoS ONE.

[46] Wang Y., Yu H.Y., Yi Z.J., Qi L.Y., Yang J.S., Xie H.X., Zhao M., Liu N.H., Chen J.Q., Zhou T.J. (2025). Extracellular vesicle–mediated mitochondrial transfer. Nat. Commun..

[47] Kitani T., Kami D., Matoba S., Gojo S. (2014). Internalization of mitochondria via macropinocytosis. J. Cell. Mol. Med..

[48] Liu Q., Liu M., Yang T., Wang X., Cheng P., Zhou H. (2023). What can we do to optimize mitochondrial transplantation therapy for myocardial ischemia-reperfusion injury?. Mitochondrion.

[49] Cowan D.B., Yao R., Thedsanamoorthy J.K., Zurakowski D., Del Nido P.J., McCully J.D. (2017). Extracellular mitochondrial integration in heart cells. Sci. Rep..

[50] Mori D., Miyagawa S., Kawamura T., Yoshioka D., Hata H., Ueno T., Toda K., Kuratani T., Oota M., Kawai K. (2023). MSC mitochondrial transfer improves cardiac function. Cell Transplant..

[51] Rossi A., Asthana A., Riganti C., Sedrakyan S., Byers L.N., Robertson J., Senger R.S., Montali F., Grange C., Dalmasso A. (2023). Mitochondrial transplantation in renal injury. Ann. Surg..

[52] Blitzer D., Guariento A., Doulamis I.P., Shin B., Moskowitzova K., Barbieri G.R., Orfany A., Del Nido P.J., McCully J.D. (2020). Delayed mitochondrial transplantation cardioprotection. Ann. Thorac. Surg..

[53] Cowan D.B., Yao R., Akurathi V., Snay E.R., Thedsanamoorthy J.K., Zurakowski D., Ericsson M., Friehs I., Wu Y., Levitsky S. (2016). Intracoronary mitochondrial delivery. PLoS ONE.

[54] Masuzawa A., Black K.M., Pacak C.A., Ericsson M., Barnett R.J., Drumm C., Seth P., Bloch D.B., Levitsky S., Cowan D.B. (2013). Transplantation of autologous mitochondria protects the heart from ischemia-reperfusion injury. Am. J. Physiol. Heart Circ. Physiol..

[55] Moskowitzova K., Shin B., Liu K., Ramirez-Barbieri G., Guariento A., Blitzer D., Thedsanamoorthy J.K., Yao R., Snay E.R., Inkster J.A.H. (2019). Mitochondrial transplantation prolongs cold ischemia time in heart transplantation. J. Heart Lung Transplant..

[56] Doulamis I.P., Guariento A., Duignan T., Orfany A., Kido T., Zurakowski D., del Nido P.J., McCully J.D. (2020). Mitochondrial transplantation for myocardial protection in diabetic hearts. Eur. J. Cardiothorac. Surg..

[57] Guariento A., Piekarski B.L., Doulamis I.P., Blitzer D., Ferraro A.M., Harrild D.M., Zurakowski D., del Nido P.J., McCully J.D., Emani S.M. (2021). Autologous mitochondrial transplantation in pediatric cardiogenic shock. J. Thorac. Cardiovasc. Surg..

[58] Baharvand F., Habibi Roudkenar M., Pourmohammadi-Bejarpasi Z., Najafi-Ghalehlou N., Feizkhah A., Bashiri Aliabadi S., Salari A., Mohammadi Roushandeh A. (2024). Platelet-derived mitochondrial transplantation in ischemic heart disease. Int. J. Cardiol..

[59] Zhang X., Yang Y., Wang H., Yan C., Feng Y., Ma X., Hu M., Li S., Cheng C. (2025). Mitochondrial health and transplantation in DCD heart transplantation. J. Transl. Med..

[60] Transplantation of Autologous Mitochondria for Ischemia-Reperfusion Injury Following ECMO Support. ICH GCP Clinical Trials Registry. https://ichgcp.net/clinical-trials-registry/NCT02851758.

[61] Autologous Mitochondrial Transplant for Cerebral Ischemia. ICH GCP Clinical Trials Registry. https://ichgcp.net/clinical-trials-registry/NCT04998357.

[62] Kim J.Y., Kang Y.C., Kim M.J., Kim S.U., Kang H.R., Yeo J.S., Kim Y., Yu S.H., Song B., Hwang J.W. (2025). Mitochondrial transplantation in inflammatory myopathy. Ann. Rheum. Dis..

[63] Phase 2 Trial of PN-101 Mitochondrial Therapy in Polymyositis/Dermatomyositis. ICH GCP Clinical Trials Registry. https://ichgcp.net/clinical-trials-registry/NCT07122648.

[64] Yamada Y., Ito M., Arai M., Hibino M., Tsujioka T., Harashima H. (2020). Challenges in mitochondrial transplantation therapy. Int. J. Mol. Sci..

[65] Ulger O., Kubat G.B. (2022). Therapeutic applications of mitochondrial transplantation. Biochimie.

[66] Roushandeh A.M., Tomita K., Kuwahara Y., Najafi-Ghalehlou N., Sato T., Roudkenar M.H. (2025). Overcoming challenges in mitochondrial transplantation. Cytotechnology.

[67] Zhang T.G., Miao C.Y. (2023). Mitochondrial transplantation therapy for mitochondrial diseases. Acta Pharm. Sin. B.

[68] Dimond R. (2015). Social and ethical issues in mitochondrial donation. Br. Med. Bull..

[69] Pickles S., Vigié P., Youle R.J. (2018). Mitophagy and Quality Control Mechanisms in Mitochondrial Maintenance. Curr. Biol..

[70] Hayakawa K., Esposito E., Wang X., Terasaki Y., Liu Y., Xing C., Ji X., Lo E.H. (2016). Transfer of mitochondria from astrocytes to neurons after stroke. Nature.

[71] Sun M., Jiang W., Mu N., Zhang Z., Yu L., Ma H. (2023). Mitochondrial transplantation as a novel therapeutic strategy for cardiovascular diseases. J. Transl. Med..

[72] Jang J., Kang K.W., Kim Y.W., Jeong S., Park J., Park J., Moon J., Jang J., Kim S., Kim S. (2024). Cardioprotection via mitochondrial transplantation. Korean J. Physiol. Pharmacol..

[73] Rosa F.L.L., de Souza I.I.A., Monnerat G., Campos de Carvalho A.C., Maciel L. (2023). Aging triggers mitochondrial dysfunction in mice. Int. J. Mol. Sci..

[74] Lejri I., Cader Z., Grimm A., Eckert A. (2024). iPSCs retain mitochondrial aging signature. Int. J. Mol. Sci..

[75] Saxena N., Taneja N., Shome P., Mani S. (2018). Mitochondrial Donation: A Boon or Curse for the Treatment of Incurable Mitochondrial Diseases. J. Hum. Reprod. Sci..

[76] Huang Y., Zhou B. (2023). Mitochondrial dysfunction in cardiac diseases and therapeutic strategies. Biomedicines.

[77] Zuccolotto Dos Reis F.H. (2024). Mitochondria and the heart. Eur. Heart J..

[78] Zhang Q., Raoof M., Chen Y., Sumi Y., Sursal T., Junger W., Brohi K., Itagaki K., Hauser C.J. (2010). Circulating mitochondrial DAMPs cause inflammatory responses to injury. Nature.

[79] Martinon F., Tschopp J. (2005). NLRs join TLRs as innate sensors of pathogens. Trends Immunol..

[80] Bahat A., MacVicar T., Langer T. (2021). Metabolism and Innate Immunity Meet at the Mitochondria. Front. Cell Dev. Biol..

[81] Gómez-Tatay L., Hernández-Andreu J.M., Aznar J. (2017). Mitochondrial Modification Techniques and Ethical Issues. J. Clin. Med..

[82] Li M., Wu L., Si H., Wu Y., Liu Y., Zeng Y., Shen B. (2025). Engineered mitochondria in diseases: Mechanisms and applications. Signal Transduct. Target. Ther..

[83] Li N., Wu B., Xiao Y., Hao Y., Wu F., Wei Y., Xu Y., Han X. (2026). Tools and delivery technologies for mitochondrial gene editing. Cell Biomater..

[84] Wong R.S.Y., Tan E.W., Goh B.H. (2026). Mesenchymal Stem Cell-Based Therapies: Challenges and Enhancement Strategies. Cell Biochem. Biophys..

[85] Qiu Y., Chang S., Zeng Y., Wang X. (2025). Advances in Mitochondrial Dysfunction and Its Role in Cardiovascular Diseases. Cells.

[86] Silva-Pinheiro P., Nash P.A., Van Haute L., Mutti C.D., Turner K., Minczuk M. (2022). In vivo mitochondrial base editing via viral delivery. Nat. Commun..

[87] Wang J., Li H., Yao Y., Zhao T., Chen Y.Y., Shen Y.L., Wang L.L., Zhu Y. (2018). Stem cell-derived mitochondrial transplantation for tissue injury. Stem Cell Res. Ther..

[88] Bacman S.R., Kauppila J.H.K., Pereira C.V., Nissanka N., Miranda M., Pinto M., Williams S.L., Larsson N.G., Stewart J.B., Moraes C.T. (2018). MitoTALEN reduces mutant mtDNA load in mice. Nat. Med..

[89] Tragni V., Primiano G., Tummolo A., Cafferati Beltrame L., La Piana G., Sgobba M.N., Cavalluzzi M.M., Paterno G., Gorgoglione R., Volpicella M. (2022). Personalized medicine in mitochondrial disease. Molecules.

